# Evidence for Dietary Agmatine Sulfate Effectiveness in Neuropathies Associated with Painful Small Fiber Neuropathy. A Pilot Open-Label Consecutive Case Series Study

**DOI:** 10.3390/nu12020576

**Published:** 2020-02-23

**Authors:** Michael L. Rosenberg, Vahid Tohidi, Karna Sherwood, Sujoy Gayen, Rosina Medel, Gad M. Gilad

**Affiliations:** 1 JFK Neuroscience Institute, JFK Medical Center, 65 James Street, Edison, NJ 08820, USA; 2 Research, Gilad&Gilad LLC, Henderson, NV 89015, USA

**Keywords:** agmatine, neuropathic pain, neuropathies, peripheral neuropathy, small fiber neuropathy

## Abstract

Peripheral neuropathies associated with painful small fiber neuropathy (SFN) are complex conditions, resistant to treatment with conventional medications. Previous clinical studies strongly support the use of dietary agmatine as a safe and effective treatment for neuropathic pain. Based on this evidence, we conducted an open-label consecutive case series study to evaluate the effectiveness of agmatine in neuropathies associated with painful SFN (Study Registry: ClinicalTrials.gov, System Identifier: NCT01524666). Participants diagnosed with painful SFN and autonomic dysfunctions were treated with 2.67 g/day agmatine sulfate (AgmaSet^®^ capsules containing G-Agmatine^®^ brand of agmatine sulfate) for a period of 2 months. Before the beginning (baseline) and at the end of the treatment period, participants answered the established 12-item neuropathic pain questionnaire specifically developed to distinguish symptoms associated with neuropathy and to quantify their severity. Secondary outcomes included other treatment options and a safety assessment. Twelve patients were recruited, and 11 patients—8 diagnosed with diabetic neuropathy, two with idiopathic neuropathy and one with inflammatory neuropathy—completed the study. All patients showed improvement in neuropathic pain to a varied extent. The average decrease in pain intensity was 26.0 rating points, corresponding to a 46.4% reduction in overall pain (*p* < 0.00001). The results suggest that dietary agmatine sulfate has a significant effect in reducing neuropathic pain intensity associated with painful SFN resistant to treatment with conventional neuropathic pain medications. Larger randomized placebo-controlled studies are expected to establish agmatine sulfate as a preferred treatment.

## 1. Introduction

Pain is a strikingly prevalent symptom in our society. It has been estimated that 20.4% of American adults have chronic pain of various etiologies [1]. Approximately half of these people have neuropathic pain secondary to small fiber neuropathy (SFN) caused by damage of small diameter somatic nerve fibers, which carry pain and temperature information, and small autonomic fibers involved in regulating sympathetic and parasympathetic nervous system functions (e.g., cardiovascular and sweat functions) [2]. This disorder is associated with many different types of neuropathy including metabolic, autoimmune, inflammatory, infectious and toxic etiologies, as well as with fibromyalgia [3]. One of the most common causes of peripheral neuropathy associated with SFN is diabetes mellitus. About half of the patients with diabetes mellitus develop peripheral neuropathy, and about one in three of these patients experience neuropathic pain [1,2]. Diagnosis in these patients is often missed as the neuropathy may precede clinical evidence of diabetes [4]. The mechanisms of diabetic neuropathic pain are still not fully clear, with both genetic and environmental factors involved [5].

Painful SFN is difficult to treat. Currently, the first line of treatment for neuropathic pain involves antidepressants such as serotonin norepinephrine reuptake inhibitors (SNRIs), or tricyclic antidepressants (TCAs) and anticonvulsants (such as gabapentin and pregabalin) [5,6,7,8]. However, overall, only about 50% pain relief is achieved in less than half of the patients treated with one of these agents, and many discontinue treatment within a few months due to poor tolerability [9,10,11,12], indicating the need for more effective treatments.

Topical therapies have had mixed results, but lidocaine, capsaicin and amitriptyline show promise [13]. Opioids have been used, but their use is controversial and may not be effective [14]. Because monotherapy in general is unsatisfactory, attempts were made to utilize combination therapies. Treatment with an anticonvulsant combined with an antidepressant, both at lower doses, may be more efficacious than either alone [15]. This also indicates that the combination treatment targets more mechanisms that may underlie the complex disease pathology [16].

There clearly is an urgent unmet need to develop safer and more effective therapies for neuropathies and neuropathic pain. To this end, we sought to assess the effectiveness of the neuroprotective dietary ingredient agmatine [17,18,19] in treating neuropathies associated with painful small fiber neuropathy.

Substantial preclinical evidence suggests the utility of agmatine in treating a wide spectrum of complex nervous system diseases [20,21]. Previous clinical trials showed that oral agmatine sulfate treatment is safe and effective in reducing neuropathic pain and improving health-related quality of life in lumbar disc-associated radiculopathy (sciatica) [22,23]. These clinical studies served as a proof-of-concept for using dietary agmatine as a nutraceutical for neuropathies.

Agmatine, decarboxylated arginine [(NH_2_(CH_2_)_4_NH_2_C(NH= )NH], is a ubiquitous molecule found in low amounts in a wide variety of plant-, fish- and animal-derived foodstuffs [24]. Additionally, gastrointestinal (GI) bacteria produce agmatine and the significant concentrations of agmatine found in the GI tract implicate microbial production as the main source of systemic agmatine [25,26]. Animal studies demonstrated that exogenous agmatine sulfate, the commonly used salt form of agmatine, is absorbed in the GI tract and then rapidly (within minutes) distributed throughout the body, including the brain [20]. In humans, ingested agmatine is readily absorbed and eliminated unmetabolized by the kidneys, with an apparent blood half-life of about 2 h [27].

Agmatine is principally metabolized into urea and putrescine, the diamine precursor of polyamines, which are essential for the viability of nerve cells [28]. Additionally, agmatine can also be oxidized, resulting in the formation of agmatine-aldehyde, which may be toxic and secreted by the kidneys [29]. This latter route is tissue specific, being significant in some tissues [26], but minor in others [30,31], and apparently negligible in the central nervous system [25].

It is postulated that, like a ‘molecular shotgun’, agmatine exerts its salutary effects by modulating multiple molecular targets including: several neurotransmitter receptors and receptor ionophores; key ionic channels and membrane transporters; nitric oxide (NO) formation; polyamine metabolism; protein ADP-ribosylation and hence signaling pathways; matrix metalloproteases (MMPs); enzymes implicated in nerve cell death and neuropathic pain; and advanced glycation end (AGE)-product formation, a process involved in the pathology of diabetes and neurodegenerative diseases [20]. Conceivably, agmatine may modulate its molecular targets both at the peripheral and the central nervous system levels.

A concerning caveat of the previous clinical trials was that after the short two-week treatment period, the effectiveness of agmatine treatment gradually dissipated [23]. This suggested that treatment should continue for as long as symptoms persist. Reports from hundreds of people who use agmatine sulfate treatment on their own cognizance (unpublished observations), support the implications of these clinical trials. Namely, the treatment is effective in alleviating symptoms in several types of neuropathy, including diabetic neuropathy and idiopathic neuropathy—which are known to involve small fiber pathology [3]—and that in order to maintain effectiveness, agmatine treatment must continue for as long as symptoms persist.

Therefore, in the present study, we set up a study to assess the effectiveness of a two-month long oral agmatine sulfate treatment for patients diagnosed with neuropathies associated with painful SFN.

## 2. Materials and Methods

### 2.1. Study Design

An open-label uncontrolled consecutive case series study was conducted to assess the therapeutic effectiveness of oral agmatine sulfate in patients with neuropathies associated with painful SFN. Therapeutic effectiveness was evaluated by measuring neuropathy-related pain sensations. Safety and tolerability were also monitored as secondary outcomes. The study was approved by the Institutional Review Boards of JFK Medical Center, Edison, NJ (Trial Number: AgS-001) and was registered with ClinicalTrials.gov Protocol Registration System (ClinicalTrials.gov, System Identifier: NCT01524666) before starting patient recruitment. The study was conducted in accordance with the Declaration of Helsinki between 2013 and 2017.

### 2.2. Study Participants

Women and men, 18 years or older, diagnosed with chronic pain secondary to SFN were considered for inclusion in the study. The type of neuropathy was established following thorough medical history review, comprehensive physical and neurological examination and laboratory analyses of blood samples. Diagnosis of SFN was confirmed by the following methods: microscopic assessment of reduced intraepidermal nerve fiber density in minimally invasive 3 mm skin biopsies [32]; and by evaluating autonomic nerve functions using the quantitative sudomotor axonal reflex test (QSART)—measuring regional ability to sweat—and the autonomic nervous system and respiration test (ANSAR) that includes measures of sympathetic and parasympathetic tone and response, orthostasis, heart rate, sympathovagal balance and breathing functions [7,33]. These have been shown to be sensitive and specific in the diagnosis of SFN [34,35]. Excluded from the study were patients who suffered from pain and numbness from causes other than SFN (such as radiculopathies and myelopathies), pregnant and breast-feeding women, and patients suffering from substance addiction.

### 2.3. Therapeutic Intervention

Eligible patients diagnosed with SFN who agreed to participate in the study read and signed the informed consent form. One day following baseline measures, patients began taking 2.67 g agmatine sulfate daily for 2 months. The treatment duration was selected based on unpublished observations of hundreds of people indicating that agmatine sulfate exerts its beneficial effects within 4 to 6 weeks and that treatment must be maintained for as long as symptoms last. Agmatine sulfate was supplied as a nutraceutical dietary supplement, AgmaSet^®^ (Gilad&Gilad LLC, Henderson, NV, USA), in size 0 gelatin capsules, each containing 445 mg of G-Agmatine^®^ brand of agmatine sulfate. AgmaSet^®^ is manufactured under cGMP (current Good Manufacturing Practice) conditions in an accredited facility in the USA. The treatment regimen consisted of 6 capsules per day (3 capsules twice daily, or 2 capsules thrice daily with or after meals). During the studies, participants could use any concomitant conventional treatments, but other experimental medications were disallowed.

### 2.4. Study Measures

Participants answered a neuropathic pain questionnaire before starting the medication (baseline) and again after the two-month treatment. The 12-item neuropathic pain questionnaire (NPQ) was developed and tested by Krause and Backonja [36] specifically for identifying and quantifying symptoms of neuropathy. It consists of questions documenting the presence and severity of the following 12 pain descriptors: burning quality, over-sensitivity to touch, shooting quality, numbness, electric sensation, tingling quality, squeezing sensation, freezing sensation, level of unpleasantness, level of overwhelming pain, increase in pain due to touch and increase in pain due to weather changes. Each question was answered on a scale of 0–100, with 100 being the most severe pain possible. Backonja and Krause [37] used these variables to calculate a total discriminant function (TDF) to distinguish patients with neuropathic pain from those without. A score of greater than 0 was predictive of neuropathic pain [37].

Safety as a secondary outcome was evaluated by analyses of clinical examinations and patients’ interviews. Tolerability was assessed based on the number of participants who failed to complete the study, of their free will or as a result of adverse effects.

### 2.5. Data Handling and Statistical Analysis

All records and data were identified by participants’ code number. All “source data” documents are stored in individual files and kept at the principal investigators’ department.

The results of each patient’s questionnaire at baseline (pretreatment) were compared with those at the end of the study. Pain descriptors were further analyzed according to Krause and Backonja [36], and changes in total discriminate function were used as an end point as well. To assess symptom improvement, data were normalized by calculating changes as percentages of baseline values. Differences were analyzed by a paired t-test and considered significant at *p* ≤ 0.05.

## 3. Results

### 3.1. Participant Characteristics

Twelve patients diagnosed with SFN were recruited to the study. One patient discontinued the treatment after a few days because “the medication had a bad taste”. No other side effect was reported and none of the participants showed any agmatine treatment-related abnormality as assessed by clinical examinations and laboratory analyses.

Table 1 summarizes the baseline demographic parameters and clinical status of the 11 participants. Five females and six males completed the study. Participants’ ages ranged from 52 to 81 years and as adjudged by body mass index (BMI) values, all were overweight (BMI values above 25) or obese (BMI values above or equal to 30). Eight participants were diagnosed with diabetic neuropathy, two with idiopathic neuropathy and one with inflammatory neuropathy. All had painful SFN based on clinical history and physical exams, confirmation by nerve fiber analysis of skin biopsies, and by either ANSAR or QSART tests, or both.

Eight of the patients had used one or more conventional neuropathic pain medications prior to starting and concurrently while taking agmatine; these medications included duloxetine, gabapentin, pregabalin, NSAIDS, topical lidocaine and tramadol (Table 1).

### 3.2. Effects of Treatment on Neuropathic Pain

#### 3.2.1. Analysis of Results for Individual Patients

Table 2 shows for each patient the average score of the 12-item NPQ before and after treatment. The absolute decrease in the average pain scores was also expressed as percentage decrease. All patients showed improvement, but to a varied extent. The average decrease in pain intensity for all 11 patients was 26.0 rating points, or a 46.4% reduction in overall pain, and was highly significant (*p* < 0.00001).

As indicated in Table 3, the calculated TDF before treatment ranged from 1.052 to 2.634 with an average of 1.731 (SD 0.44), strongly supporting that the pain was neuropathic in origin. At the completion of treatment, TDF decreased in all but one patient, and ranged from −1.123 to 1.812 with an average of 0.319 (SD 0.32).

#### 3.2.2. Analysis of Results by Pain Category

Table 4 shows the mean pain rating values recorded by all study participants before and after treatment and the average decrease in pain levels for each of the NPQ 12 pain descriptors. The average decrease in all pain categories was 26 rating points, ranging from a minimum of 16.7 for squeezing pain to 38.3 for burning pain; this corresponds to a 46.4% reduction (significant at *p* < 0.0001). Numbness, tingling and burning, the symptoms most clinically associated with neuropathic pain, were the most highly rated at the onset of the study with a mean pain level of more than 70 rating points. These three symptoms also showed the greatest reductions after treatment with agmatine, by an order of magnitude, with an average decrease greater than 31.6 rating points after treatment (significant at *p* < 0.001). Reductions in the electric, squeezing and increased pain due to touch categories did not reach statistical significance (Table 4).

## 4. Discussion

The results of the present study provide evidence that a two-month treatment with the neuroprotective dietary ingredient agmatine sulfate is effective in alleviating neuropathic pain in patients suffering from neuropathies associated with painful SFN. The results indicate that agmatine treatment should continue for as long as symptoms persist and corroborate previous observations [22,23] showing that dietary agmatine sulfate treatment lacks any significant side effects. The findings also lend support to unpublished observations of hundreds of people who are, on their own cognizance, using long-term (years) agmatine sulfate treatment for various types of neuropathy involving SFN.

All participants who entered this open-label consecutive case series study had neuropathy associated with SFN as adjudged by reduced numbers of nerve fibers in skin biopsies and by abnormal autonomic nerve functions using the ANSAR and QSART [7,32,33,34,35]. The painful symptoms in all patients were confirmed to be neuropathic using accepted criteria of the 12-item questionnaire (NPQ) and calculated changes in pain descriptors (TDF), which distinguish neuropathic pain from other types of pain according to Krause and Backonja [36,37].

The symptoms most clinically associated with neuropathic pain numbness, tingling and burning [36,37] showed the greatest response to treatment with agmatine, suggesting that these neuropathic pain descriptors are associated with SFN involving autonomic nerves. Reductions in the categories least considered characteristic of neuropathic pain—electric, squeezing and increased pain due to touch [36,37]—did not reach statistical significance after agmatine treatment.

Ample evidence indicates that agmatine sulfate can modulate multiple molecular targets implicated in neuroprotection and in mitigating neuropathic pain [20]. These include modulation of key neurotransmitter receptors [including nicotine, N-methyl-D-aspartate (NMDA), imidazoline and α2-adrenoceptors], ionic channels (including potassium and calcium channels), cell signaling pathways (by inhibiting ADP-ribosylation of proteins), nitric oxide (NO) synthesis, polyamine metabolism and extracellular protein modifications (by inhibiting matrix metalloproteases and advanced glycation end (AGE)-product formation) [20]. The spectrum of molecular mechanisms underlying painful SFN may be even broader [38]. With this body of evidence taken together with the fact that anti-inflammatory drugs are ineffective [4,5,6,7,8], the results of the present study further validate the notion that agmatine exerts its salutary action on nervous system-associated processes, rather than by acting on inflammatory mechanisms [20].

Peripheral neuropathies associated with painful SFN are complex, difficult to treat conditions, which often require a drug combination treatment. The first line of treatment involves antidepressants such as SNRIs (serotonin norepinephrine reuptake inhibitors), or TCAs (tricyclic antidepressants) and anticonvulsants (such as gabapentin and pregabalin) [4,5,6,7,8]. Interestingly, substantial preclinical evidence indicates that agmatine treatment exerts antidepressant and anti-seizure effects in animal models of depression and epilepsy, respectively [20,21]. Additionally, one clinical case study reported the antidepressant effects of oral agmatine sulfate in three patients [39]. Based on the cumulative evidence, it is postulated that ingested agmatine sulfate exerts its beneficial effects by interacting simultaneously like a ‘molecular shotgun’ with multiple molecular mechanisms critical for neuropathic pain [23]. Agmatine is readily absorbed [26,40] and may modulate these molecular targets in both the central and peripheral nervous systems [41].

While the results of this study are encouraging, there are several major limitations, as follows. (1) It was an open-label uncontrolled study. For example, a randomized placebo-controlled study would account for the possible effects of concomitant treatments. (2) It consisted of a small sample size. For example, a larger sample would enable the assessment of gender, age or BMI differences. (3) It did not employ objective follow-up measures such as skin biopsies, ANSAR and QSART measures to assess whether neuropathic pain improvement measures are associated with the structure and function recovery of small nerve fibers; in this regard, longer follow-up periods than the two-month period used in the present study will be required.

In summary, this pilot study suggests that agmatine sulfate has a significant effect in reducing overall pain intensity in patients with SFN resistant to treatment with conventional neuropathic pain medications. The inadequate effectiveness of current pharmacotherapy underscores the importance of the continued research and development into agmatine as a novel treatment for neuropathies. Further randomized placebo-controlled studies, conducted over properly extended periods with adequate numbers of participants, are required to establish agmatine sulfate as a preferred treatment.

## Figures and Tables

**Table 1:** Baseline demographic parameters and clinical status.

Patient Number	Gender	Age (Years)	BMI ^1^	Neuropathy Type	Skin Biopsy ^2^	Autonomic Functions		Concurrent Pain Medications
						QSART ^3^	ANSAR ^4^	
**1**	Female	81	39.13	Diabetic	Abnormal	Normal	Abnormal	Duloxetine
**2**	Male	69	39.31	Diabetic	Abnormal	Abnormal	Abnormal	Gabapentin, Indomethacin
**3**	Male	64	33.25	Diabetic	Abnormal	Abnormal	Abnormal	Duloxetine, Gabapentin, Topical Lidocaine
**4**	Male	77	28.13	Idiopathic	Abnormal	Lost Record	Lost Record	Gabapentin, Pregabalin, Tramadol, Cyclobenzaprin, Topical Lidocaine
**5**	Male	57	27.13	Diabetic	Abnormal	Abnormal	Normal	None
**6**	Female	52	31.00	Diabetic	Abnormal	Abnormal	Abnormal	Amitriptyline, Pregabalin, Baclofen
**7**	Male	58	27.40	Diabetic	Abnormal	Abnormal	Normal	None
**8**	Male	61	35.13	Diabetic	Abnormal	Abnormal	Abnormal	Gabapentin, Meloxicam
**9**	Female	56	27.22	Inflammatory	Abnormal	Normal	Abnormal	None
**10**	Female	55	35.56	Diabetic	Abnormal	Abnormal	Abnormal	Celecoxib, Tramadol
**11**	Female	58	27.20	Idiopathic	Abnormal	Normal	Abnormal	Pregabalin

^1^ BMI values are calculated as kg/m^2^ (weight in kg divided by the square of height in meters). ^2^ Abnormal Skin Biopsy means reduced nerve fiber density. ^3^ Abnormal QSART means reduced sweat functions. ^4^ Abnormal ANSAR means reduced measures of autonomic nervous system functions. QSART, quantitative sudomotor axonal reflex test; ANSAR, autonomic nervous system and respiration test; BMI, body mass index.

**Table 2:** Average values of pain ratings for each patient before and after treatment with agmatine sulfate. The differences between average rating point values are also expressed as percentages ^1^.

Patient	Average Pain Before Treatment	Average Pain After Treatment	Absolute Decrease (Rating Points)	Percent Decrease
1	78.3	44.2	34.1	43.6%
2	64.2	59.1	5.1	29.2%
3	55.6	34.2	21.4	38.5%
4	38.3	35.8	2.5	6.5%
5	71.3	6.7	64.7	90.7%
6	83.3	2.3	81	97.2%
7	60	47.5	12.5	20.8%
8	48.3	40.0	8.3	17.2%
9	15.8	11.7	4.2	26.3%
10	45.3	34.2	11.1	24.4%
11	56.7	28.7	28	49.3%
**Average (SD)**	**56.1 (18.2)**	**30.1 (16.9)**	26.0 *	46.4% *

^1^ Average pain represents raw values of the 12-item neuropathic pain questionnaire (NPQ) rating on a scale of 0-100. * Average of all 11 patient values was highly significant at *p* < 0.00001 (paired *t*-test). SD, standard deviation.

**Table 3:** Total discriminant function (TDF) score for each subject before and after two months of agmatine sulfate treatment, also showing average values.

Patient Number	Before Treatment	After Treatment
1	1.477	−0.138
2	1.632	0.852
3	1.992	−1.138
4	1.052	0.272
5	1.162	0.532
6	2.634	−1.123
7	1.562	1.812
8	2.009	0.731
9	1.933	0.691
10	1.862	0.707
11	2.022	0.337
Average (SD)	1.731 (0.44)	0.3198 (0.32) *

* Significant at a level of *p* < 0.0001 (paired *t*-test). SD, standard deviation.

**Table 4:** Mean pain ratings before and after treatment of all participants and the average decreases in pain levels for the 12 pain descriptors (categories) of the neuropathic pain questionnaire (NPQ) ^1^.

Pain Category	Mean Pain Ratings Before Treatment	Mean Pain Ratings After Treatment	Average Decrease in Pain Levels (SD)	*p* Value *
Burning	70.8	32.5	38.3 (28.8)	0.0008
Oversensitivity to Touch	46.4	20.9	25.5 (40.1)	0.022
Shooting Pain	62.1	30.8	31.3 (40.3)	0.01
Numbness	76.4	44.7	31.7 (323)	0.001
Electric	52.5	34.1	18.4 (43.3)	0.11
Tingling	76.4	43.4	33.0 (30.3)	0.002
Freezing	41.0	23.6	17.4 (403)	0.038
Unpleasantness	69.9	40.5	29.4 (35.4)	0.01
Overwhelming	65.0	37.0	28.0 (37.2)	0.015
Squeezing	31,8	15.1	16.7 (45.3)	0.194
Increased Pain Due to Touch	42.9	22.3	20.6 (36.7)	0.27
Increased Pain Due to Weather Changes	38.1	15.9	22.2 (35.7)	0.021

^1^ Mean pain ratings represent raw values of the 12-item neuropathic pain questionnaire (NPQ) on a scale of 0-100. Scores of the 12 types of pain were averaged. * *p* ≤ 0.05 was considered significant (paired *t*-test). SD, standard deviation.
